# THE IMPORTANCE OF ADEQUATE RESOURCES AND EQUIPMENT TO STAFF ENGAGEMENT IN A HEALTHCARE SYSTEM

**DOI:** 10.1093/geroni/igad104.3647

**Published:** 2023-12-21

**Authors:** Orah Burack, Wingyun Mak, Margret Howard, Tanya Isaacs, Joann Reinhardt

**Affiliations:** The New Jewish Home Research Institute on Aging, New York City, New York, United States; The New Jewish Home Research Institute on Aging, New York City, New York, United States; The New Jewish Home Research Institute on Aging, New York City, New York, United States; The New Jewish Home, New York City, New York, United States; The New Jewish Home, New York City, New York, United States

## Abstract

During this time of unprecedented staffing challenges, identifying factors impacting staff engagement may be one step towards rebuilding the healthcare workforce. This study was designed to identify work factors differentiating positively engaged employees from others. In May 2023, 350 employees (response rate=30%) from across a healthcare system (including skilled nursing facilities, adult day centers, community services, and assisted living) serving older adults in New York City and Westchester County, completed surveys examining employee engagement and satisfaction with five work factors. Engagement was measured using the employee net promoter scale (eNPS) in which employees rate from 1 (Not At All) to 10 (Very) “How likely is it that you would recommend the organization as a workplace.” Those answering 9-10 are classified as “Promoters” (engaged/positive concerning the workplace), 7-8 “Neutrals”, and 1-6 are “Detractors” (unengaged/negative). Participants also responded 1 “very unsatisfied” to 5 “very satisfied” on work factors: general work satisfaction, having enough resources/equipment, receiving timely information, supervisor support, and coworker respect. Based on eNPS responses, 160 (45.7%) employees were “Promoters”, 65 (18.6%) “Neutrals”, and 125 (35.7%) “Detractors.” A series of one-way analysis of variance and post-hoc Bonferonni analyses then examined work factor satisfaction by staff engagement level. As expected, “Promoters” and “Neutrals” were significantly more satisfied than “Detractors” on all five work factors however, only having enough resources/equipment separated “Promoters” (M=3.63, SD=1.24) from “Neutrals” (M=4.06, SD=89, p <.05). These findings indicate that providing staff with sufficient resources/equipment should be prioritized in the effort to establish a healthcare workforce of “Promoters.”

